# The clinical predictive value of geriatric nutritional risk index in elderly rectal cancer patients received surgical treatment after neoadjuvant therapy

**DOI:** 10.3389/fnut.2023.1237047

**Published:** 2023-08-21

**Authors:** Lei Zhang, Chenhao Hu, Ruizhe Li, Zhe Zhang, Ya Wang, Jiamian Zhao, Ruihan Liu, Zhenghui Li, Junjun She, Feiyu Shi

**Affiliations:** ^1^Department of General Surgery, The First Affiliated Hospital of Xi’an Jiaotong University, Xi’an, Shaanxi, China; ^2^Center for Gut Microbiome Research, Med-X Institute, The First Affiliated Hospital of Xi’an Jiaotong University, Xi’an, Shaanxi, China

**Keywords:** elderly patients, rectal cancer, geriatric nutritional risk index, postoperative major complication, cancer-specific survival, neoadjuvant therapy

## Abstract

**Objective:**

The assessment of nutritional status has been recognized as crucial in the treatment of geriatric cancer patients. The objective of this study is to determine the clinical predictive value of the geriatric nutritional risk index (GNRI) in predicting the short-term and long-term prognosis of elderly rectal cancer (RC) patients who undergo surgical treatment after neoadjuvant therapy.

**Methods:**

Between January 2014 and December 2020, the clinical materials of 639 RC patients aged ≥70 years who underwent surgical treatment after neoadjuvant therapy were retrospectively analysed. Propensity score matching was performed to adjust for baseline potential confounders. Logistic regression analysis and competing risk analysis were conducted to evaluate the correlation between the GNRI and the risk of postoperative major complications and cumulative incidence of cancer-specific survival (CSS). Nomograms were then constructed for postoperative major complications and CSS. Additionally, 203 elderly RC patients were enrolled between January 2021 and December 2022 as an external validation cohort.

**Results:**

Multivariate logistic regression analysis showed that GNRI [odds ratio = 1.903, 95% confidence intervals (CI): 1.120–3.233, *p* = 0.017] was an independent risk factor for postoperative major complications. In competing risk analysis, the GNRI was also identified as an independent prognostic factor for CSS (subdistribution hazard ratio = 3.90, 95% CI: 2.46–6.19, *p* < 0.001). The postoperative major complication nomogram showed excellent performance internally and externally in the area under the receiver operating characteristic curve (AUC), calibration plots and decision curve analysis (DCA). When compared with other models, the competing risk prognosis nomogram incorporating the GNRI achieved the highest outcomes in terms of the C-index, AUC, calibration plots, and DCA.

**Conclusion:**

The GNRI is a simple and effective tool for predicting the risk of postoperative major complications and the long-term prognosis of elderly RC patients who undergo surgical treatment after neoadjuvant therapy.

## 1. Introduction

Population ageing is increasing worldwide. According to the recent Demographic Statistics Report, the population older than 70 is expected to double by 2050 (1). While increased life expectancy is considered a positive phenomenon, it also means vulnerability to diseases such as cancer, which is currently one of the leading causes of death in the world (2). Rectal cancer (RC), ranking eighth in global morbidity, predominantly affects older adults, and its incidence has increased in recent years (3). At present, neoadjuvant therapy followed by total mesorectal excision is routinely recommended for locally advanced RC (4).

However, due to the toxic side effects of chemoradiotherapy, adverse nutritional status is more common in RC patients receiving neoadjuvant therapy (5). In addition, elderly cancer patients are more prone to suffer from malnutrition owing to multiple factors such as comorbid illnesses, immunosenescence, organ decline, and increased energy expenditure caused by the tumour. According to previous studies, malnutrition was associated with cancer treatment intolerance and postoperative complications, seriously affecting the long-term survival of patients (6–8). Therefore, preoperative nutritional screening in elderly patients with RC can help identify high-risk individuals who may benefit from targeted interventions to improve prognosis and enhance quality of life.

The geriatric nutritional risk index (GNRI) was initially proposed by Bouillanne et al. as an assessment measure to identify elderly medical patients who are at risk of malnutrition and related mortality (9). The GNRI can be calculated using routine haematological parameters such as height, weight, and albumin, and its use can help save physicians’ time during the pretherapeutic evaluation. Numerous recent studies have shown that GNRI is not only a valuable nutritional assessment tool for chronic diseases but also for predicting clinical outcomes in malignant tumours (10–13).

So far, there have been limited studies investigating the relationship between GNRI and RC patients who have undergone neoadjuvant therapy. Furthermore, no study has explored the association between the GNRI and the short-term prognosis of RC. Hence, the objective of this study is to assess the clinical predictive value of preoperative GNRI levels for postoperative major complications and cancer-specific survival (CSS) in RC patients over 70 years of age who have received surgical treatment following neoadjuvant therapy.

## 2. Methods

### 2.1. Study cohort

The clinical data of 955 elderly RC patients who underwent neoadjuvant therapy and surgery between January 2014 and December 2020 were collected from the prospective cancer database of the Department of General Surgery in our institution. Exclusion criteria were as follows: (i) underwent open surgery; (ii) with yp stage IV; (iii) without relevant clinicopathological information or survival status; and (iv) underwent palliative therapy. Patients from January 2021 to December 2022 were included as the external validation cohort. A flow chart of the patients included in the study is presented in Supplementary Figure S1. All patients provided written informed consent for the collection of data and subsequent analyses. The Institutional Review Board and Ethical Committee of the First Affiliated Hospital of Xi’an Jiaotong University approved this study (XJTU1AF2020LSL-004).

### 2.2. Data collection and follow-up

Clinicopathological parameters were collected from patients, including age, sex, American Society of Anaesthesiologists (ASA) score, tumour size, tumour location, differentiation grade, histological type, surgical approach, tumour-node-metastasis (TNM) stage, neoadjuvant treatment, and tumour regression grade (TRG). The Eighth Edition of the American Joint Committee on Cancer (AJCC) Staging for colorectal cancer was applied to identify the TNM stage system (14). According to the scoring system developed by the AJCC, the range of TRG after neoadjuvant therapy is 0 to 3. We defined TRG 0 and 1 as “good response” and TRG 2 and 3 as “poor response.” Data regarding the postoperative complications and recovery of all patients was collected. Venous blood was drawn from all patients within 3 d before surgery. Preoperative routine blood examination results [such as albumin, carcinoembryonic antigen (CEA), and CA19-9, etc.] were also recorded.

In our institution, all patients adopted similar follow-up routines. Reexaminations, either telephone or outpatient clinic, were recommended every 3 months within 2 years after surgery and then every 6 months thereafter. The last follow-up date in this study was 31 January 2023.

### 2.3. Definition of the GNRI

The definition of the GNRI was described in previous studies (15). The GNRI was calculated using the following formula: GNRI = 14.89 × albumin (g/dl) + 41.7 × usual weight/ideal weight. Ideal weight was calculated by the Lorentz formula: male = 0.75 × height (cm) – 62.5 and female =0.60 × height (cm) – 40. Next, X-tile software was used to determine and visualize the best cutoff points of the GNRI in this study. According to the x-tile software, the best GNRI cutoff value for predicting CSS was 94.6 (Supplementary Figure S2). Thus, the cohort of patients was divided into a low-level GNRI group (GNRI, < 94.6) and a normal GNRI group (GNRI, ≥ 94.6).

### 2.4. Propensity score matching analysis

In this study, the enrolled patients were divided in terms of GNRI levels rather than at random; thus, selection bias and confounding factors would diminish the reliability of the results. Thus, propensity score matching (PSM) of the two groups was conducted to minimize selection bias. Age, sex, ASA score, tumour size, tumour location, differentiation grade, histological type, surgical approach, yp TNM stage, neoadjuvant treatment, and TRG were identified as match parameters. The methodology used for PSM is further described in the Supplementary Materials.

### 2.5. Outcomes and covariables

The short-term outcome focused on evaluating the connection between preoperative GNRI level and postoperative major complications, defined as Clavien–Dindo (CD) grade ≥ II (16). The long-term outcome aimed to assess the relationship between preoperative GNRI level and CSS in elderly RC patients.

### 2.6. Statistical analysis

All analyses were performed with IBM SPSS Statistics 26.0 software, R version 4.1.0 and the X-tile program. Two-tailed *p* values <0.05 were assessed as statistically significant. The optimal cutoff values of the GNRI were estimated by using the X-tile program (17). Categorical variables were compared using the *χ*^2^ test or Fisher’s exact test. Continuous variables were compared using the Mann–Whitney *U* test. Univariate and multivariate logistic analyses were used to evaluate the risk factors for postoperative major complications. Competing risk analysis was performed to find the associations between factors and rectal cancer-specific mortality. The methodology of competing risk analysis is further described in the Supplementary Materials. According to the independent risk factors screened by multivariate analysis, a postoperative complication prediction nomogram and several CSS prediction nomograms were constructed. The receiver operating characteristic (ROC) curve, calibration curve, concordance index (C-index), and decision curve analysis (DCA) were applied to evaluate the clinical application of the nomograms.

## 3. Results

### 3.1. Patient characteristics

A total of 639 elderly RC patients (≥ 70 years) were selected for the modelling cohort, each undergoing neoadjuvant therapy and radical resection. Among them, 207 patients (32.4%) and 432 (67.6%) patients were in the low-level GNRI (< 94.6) and normal GNRI (≥ 94.6) groups, respectively. The enrolled patients’ baseline characteristics of the two groups before and after PSM are outlined in Table 1. In single-factor analysis, at low-level (vs. normal) GNRI, age was significantly higher (78.9 ± 4.2 vs. 76.2 ± 5.1, *p* < 0.001), the proportion of women was significantly increased (50.8% vs. 45.8%, *p* = 0.050), the proportion of ASA score III–IV was significantly higher (49.8% vs. 37.7%, *p* = 0.004), and the proportion of TNM III stage was significantly increased (55.6% vs. 44.9%, *p* = 0.002). Regarding other measured variables, there was no significant difference between those two groups (all *p* > 0.05). After 1:1 PSM, the clinical variables, including age, sex, ASA score, tumour size, tumour location, differentiation grade, histological type, surgical approach, TNM stage, neoadjuvant treatment and TRG, were well balanced between the two groups (all *p* < 0.05). The basic characteristics of the validation cohort are shown in Supplementary Table S1.

**Table 1:** Baseline characteristics of enrolled elderly rectal cancer patients received surgical treatment after neoadjuvant chemotherapy before and after propensity score matching.

Variable	Before matching		*p*-value	After matching		*p*-value
	Low-level GNRI (*n* = 207)	Normal GNRI (*n* = 432)		Low-level GNRI (*n* = 192)	Normal GNRI (*n* = 192)	
Age, yr	78.9 ± 4.2	76.2 ± 5.1	<0.001	78.1 ± 4.7	77.6 ± 5.9	0.541
Sex			0.050			0.473
Male	95 (45.9)	234 (54.2)		91 (47.4)	84 (43.8)	
Female	112 (54.1)	198 (45.8)		101 (52.6)	108 (56.2)	
ASA score			0.004			0.540
I or II	104 (50.2)	269 (62.3)		95 (49.5)	101 (52.6)	
III or IV	103 (49.8)	163 (37.7)		97 (50.5)	91 (47.4)	
Tumor size, mm			0.865			0.788
<50	114 (55.1)	241 (55.8)		103 (53.6)	100 (52.1)	
>50	93 (44.9)	191 (44.2)		89 (46.4)	92 (47.9)	
Distance to the anal verge, cm			0.846			0.928
11–15	57 (27.5)	121 (28.0)		52 (27.1)	55 (28.6)	
6–10	72 (34.8)	158 (36.6)		70 (36.5)	67 (34.9)	
0–5	78 (37.7)	153 (35.4)		70 (36.5)	70 (36.5)	
Differentiation grade			0.274			0.444
Well or moderate	179 (86.5)	359 (83.1)		170 (88.5)	165 (85.9)	
Poor or worse	28 (13.5)	73 (16.9)		22 (11.5)	27 (14.1)	
Histology			0.269			0.528
Adenocarcinoma	153 (73.9)	301 (69.7)		150 (78.1)	155 (80.7)	
Mucinous adenocarcinoma or signet-ring cell	54 (26.1)	131 (30.3)		42 (21.9)	37 (19.3)	
Surgical approach			0.787			0.812
Laparoscopic surgery	160 (77.3)	338 (78.2)		146 (76.0)	144 (75.0)	
Robotic surgery	47 (22.7)	84 (21.8)		46 (24.0)	48 (25.0)	
yp TNM stage			0.002			0.567
I	21 (10.1)	89 (20.6)		19 (9.9)	23 (12.0)	
II	71 (34.3)	149 (34.5)		68 (35.4)	74 (38.5)	
III	115 (55.6)	194 (44.9)		105 (54.7)	95 (49.5)	
Neoadjuvant treatment			0.448			0.913
RT	64 (30.9)	121 (28.0)		61 (31.8)	60 (31.3)	
CRT	143 (69.1)	311 (72.0)		131 (68.2)	132 (68.7)	
TRG			0.130			0.679
Good response	96 (46.4)	228 (52.8)		83 (43.2)	79 (41.1)	
Poor response	111 (53.6)	204 (47.2)		109 (56.8)	113 (58.9)	

GNRI, geriatric nutritional risk index; ASA, American society of Aneshesiologists; CRT, chemoradiotherapy; RT, radiotherapy; TRG, tumour regression grade.

### 3.2. The association of preoperative GNRI level and perioperative outcomes

The association of different GNRI levels and perioperative outcomes is shown in Table 2. The low-level GNRI group was associated with significantly higher rates of overall complications (33.3% vs. 21.4%, *p* = 0.008) and major complications (27.6% vs. 17.2%, *p* = 0.014). Moreover, the rate of anastomotic leakage was significantly higher in the low-level GNRI group than in the normal GNRI group (7.3% vs. 2.6%, *p* = 0.034). In terms of postoperative recovery, there were no significant differences observed between the two groups in the time to first flatus, time to tolerate liquid diets and length of postoperative hospital stay.

**Table 2:** The association of GNRI and perioperative outcomes in elderly rectal cancer patients received surgical treatment after neoadjuvant chemotherapy.

Variables	Low-level GNRI (*n* = 192)	Normal GNRI (*n* = 192)	*p*-value
Operation time (min), median (range)	210 (150–500)	220 (130–420)	0.472
Estimated blood loss (mL), median (range)	120 (50–500)	110 (20–400)	0.394
Postoperative complications	64 (33.3)	41 (21.4)	0.008
Clavien-Dindo grade ≥ II	53 (27.6)	33 (17.2)	0.014
Surgical site infection	12 (6.3)	8 (4.2)	0.358
Ileus	5 (2.6)	5 (2.6)	1.000
Anastomotic leakage	14 (7.3)	5 (2.6)	0.034
Intra-abdominal abscess	7 (3.6)	6 (3.1)	0.778
Cardiovascular events	7 (3.6)	3 (1.6)	0.200
Pneumonia	7 (3.6)	5 (2.6)	0.557
Urinary infection	1 (0.5)	1 (0.5)	1.000
Days to first flatus (days), median (range)	3 (1–11)	2 (1–6)	0.178
Days to soft diet (days), median (range)	5 (2–16)	4 (2–13)	0.156
Postoperative hospital stay (days), median (range)	11 (6–59)	9 (3–25)	0.221
Mortality	2 (1.0)	0 (0)	0.478

GNRI, geriatric nutritional risk index.

### 3.3. Risk factors for CD grade ≥ 2 postoperative complications

To select the best variables for the postoperative major complication predictive model, we performed a risk factor analysis (Table 3). Univariate analysis indicated that age ≥ 80 years, low-level GNRI, ASA score III–IV, laparoscopic surgery and clinical T stage were risk factors for CD grade ≥ 2 postoperative complications (all *p* < 0.05). Further multivariate logistic analysis results revealed that age ≥ 80 years [odds ratio (OR) = 2.107, 95% confidence interval (CI): 1.250–3.553, *p* = 0.005], preoperative low-level GNRI (OR = 1.903, 95% CI: 1.120–3.233, *p* = 0.017), robotic surgery (OR = 0.364, 95% CI: 0.195–0.678, *p* = 0.001) and clinical T stage (OR = 2.985, 95% CI: 1.752–5.086, *p* < 0.001) were independent risk factors for CD grade ≥ 2 postoperative complications.

**Table 3:** Univariate and multivariate analyses of predictors for postoperative complications (Clavien-Dindo grade ≥ II) in elderly rectal cancer patients received surgical treatment after neoadjuvant chemotherapy.

Variables	Univariate analysis		Multivariate analysis	
	Odds ratio (95 CI)	*p*-value	Odds ratio (95 CI)	*p*-value
Age		0.003		0.005
<80	Reference		Reference	
≥80	2.098 (1.284–3.426)		2.107 (1.250–3.553)	
Sex		0.086		
Male	Reference			
Female	0.653 (0.401–1.062)			
GNRI		0.031		0.017
≥94.6	Reference		Reference	
<94.6	1.721 (1.050–2.815)		1.903 (1.120–3.233)	
ASA score		0.016		0.080
I or II	Reference		Reference	
III or IV	1.839 (1.123–3.011)		1.597 (0.945–2.697)	
Tumor size, mm		0.563		
<50	Reference			
>50	1.154 (0.711–1.874)			
Distance to the anal verge, cm		0.674		
11–15	Reference			
6–10	1.045 (0.573–1.906)			
0–5	1.282 (0.718–2.289)			
Differentiation grade		0.118		
Well or moderate	Reference			
Poor or worse	1.630 (0.883–3.099)			
Histology		0.772		
Adenocarcinoma	Reference			
Mucinous adenocarcinoma or signet-ring cell	1.010 (0.590–2.406)			
Surgical approach		0.005		0.001
Laparoscopic surgery	Reference		Reference	
Robotic surgery	0.379 (0.209–0.684)		0.364 (0.195–0.678)	
Clinical T stage		<0.001		<0.001
T1-2	Reference		Reference	
T3-4	3.038 (1.820–5.071)		2.985 (1.752–5.086)	
Clinical N stage		0.694		
N0	Reference			
N1-2	1.103 (0.678–1.792)			
Neoadjuvant treatment		0.764		
RT	Reference			
CRT	0.928 (0.572–1.506)			
TRG		0.532		
Good response	Reference			
Poor response	0.857 (0.528–1.391)			
CEA		0.309		
Normal	Reference			
High	1.287 (0.791–2.094)			
CA19-9		0.765		
Normal	Reference			
High	1.077 (0.664–1.748)			

GNRI, geriatric nutritional risk index; ASA, American society of Aneshesiologists; CRT, chemoradiotherapy; RT, radiotherapy; TRG, tumour regression grade.

### 3.4. Postoperative complication nomogram development and validation

**Figure 1:**
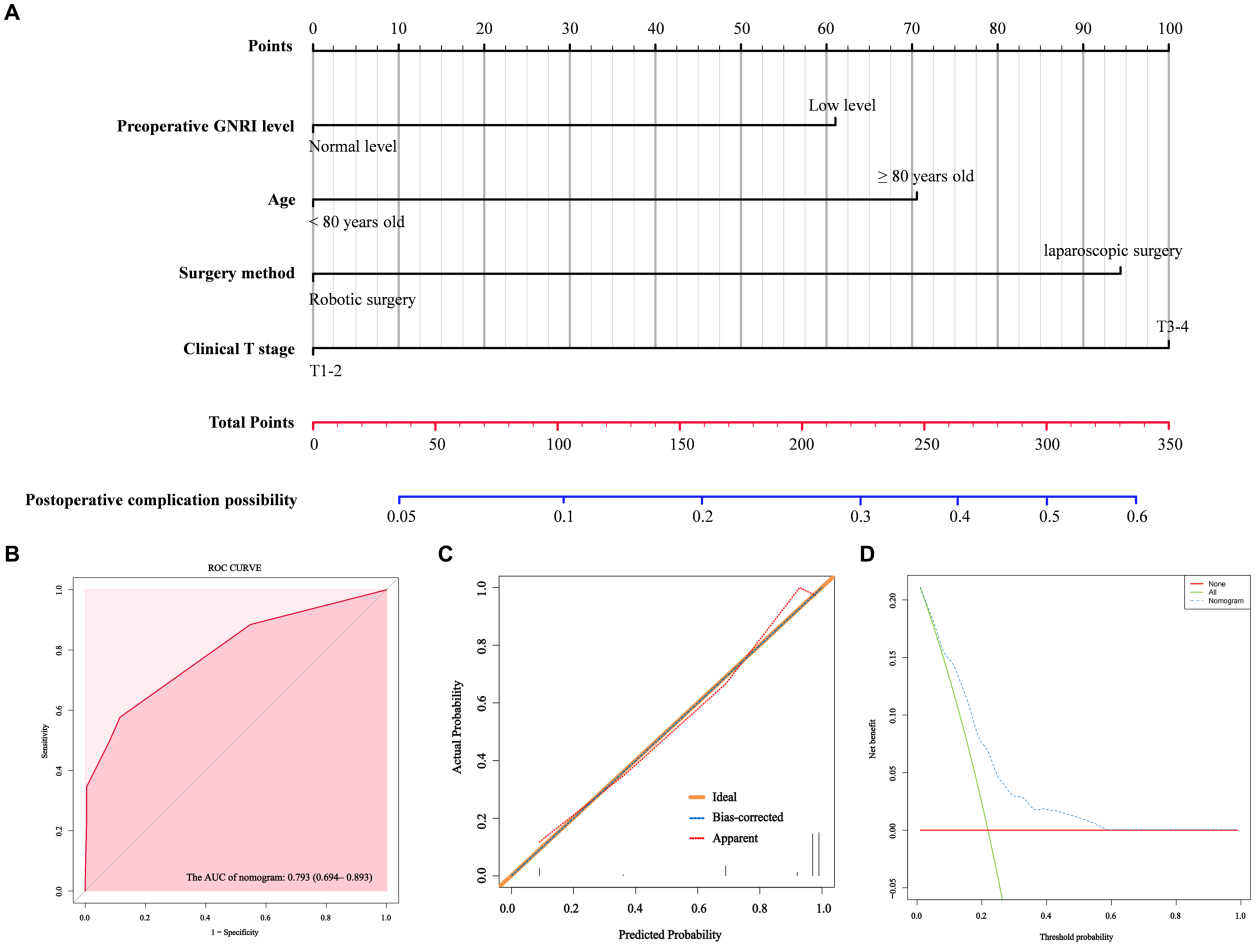
A nomogram model for predicting the risk of postoperative major complications in elderly rectal cancer patients underwent surgical treatment after neoadjuvant therapy **(A)**. ROC curve **(B)**, calibration curves **(C)** and DCA curves **(D)** for the model.

**Figure 2:**
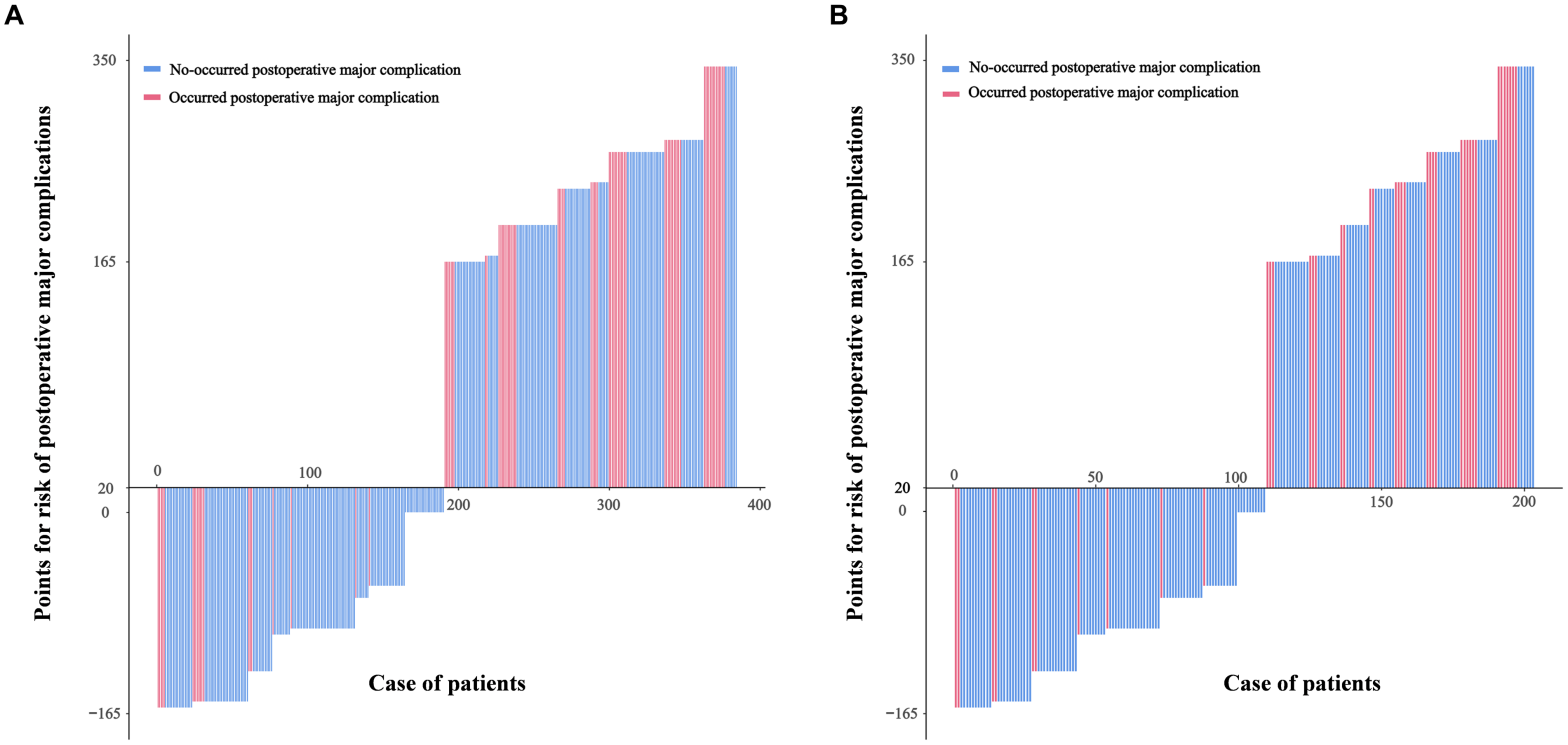
Patients in the development cohort **(A)** and the validation cohort **(B)** were divided into low-risk and high-risk groups based on the cutoff value of the total score of the nomogram.

According to the results of multivariate analysis, we constructed a nomogram to predict postoperative major complications (Figure 1A). The area under the curve (AUC) of this nomogram was 0.793 (95% CI, 0.694–0.893), and the calibration plot observed and predicted results were in good agreement (Figures 1B,C). Additionally, the DCA curves illustrated that the nomogram had good clinical utility (Figure 1D). In the validation cohort, the nomogram also displayed an AUC of 0.724 (95% CI, 0.666–0.781) with good predictive efficacy in the calibration plot and DCA plot (Supplementary Figure S3). To better evaluate the predictive effect of the nomogram, we carried out risk classification according to the nomogram cutoff value (165 points) determined by the maximum Youden index of the nomogram ROC curve. The application of this cutoff value to the development cohort [47.2% (high risk group) vs. 13.4% (low risk group), *p* < 0.001] and validation cohort [33.7% (high risk group) vs. 10.5% (low risk group), *p* < 0.001] showed good discrimination in the postoperative major complications (Figures 2A,B).

### 3.5. The association of preoperative GNRI level and prognosis

**Figure 3:**
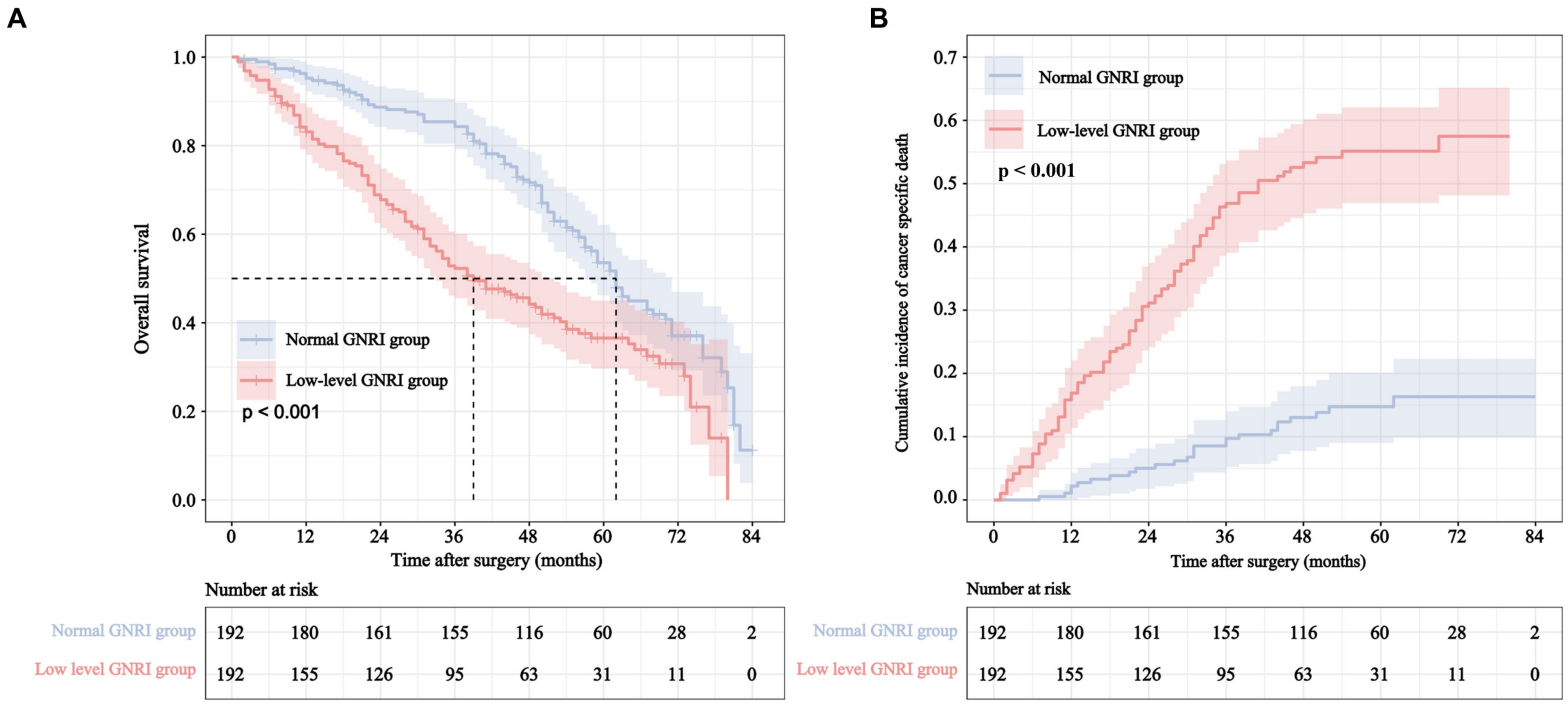
Overall survival curve **(A)** and cumulative incidence curve of cancer specific mortality **(B)** for different GNRI levels in elderly rectal cancer patients underwent surgical treatment after neoadjuvant therapy.

The cumulative 5-year incidence of rectal overall survival (OS) and CSS in the low-level GNRI group were 36.5 and 44.7%, respectively, which were significantly worse than those in the normal GNRI group (53.6 and 83.9%, *p* < 0.001 and *p* < 0.001, respectively) (Figure 3). We further performed stratified analysis according to whether patients were over 80 years old. Compared with the normal GNRI group, the low-level GNRI group exhibited worse 5-year OS (46.3% vs. 58.1%, *p* = 0.008) and CSS (49.8% vs. 89.7%, *p* < 0.001) in 70- to 80-year-old patients (Supplementary Figures S4A,B). Analogously, patients aged > 80 years in the low GNRI group had worse 5-year OS (23.4% vs. 49.1%, *p* < 0.001) and CSS (38.9% vs. 78.7%, *p* < 0.001) than those in the normal GNRI group (Supplementary Figures S5A,B).

### 3.6. Prognostic variables for rectal cancer specific mortality

Table 4 shows the results of competing risk analysis. Univariate and multivariate competing risk analyses indicated that the factors of age > 80 years [subdistribution hazard ratio (SHR) = 1.76, 95% CI: 1.14–2.74, *p* = 0.012], GNRI <94.6 (SHR = 3.90, 95% CI: 2.46–6.19, *p* < 0.001), poorly differentiated or undifferentiated (SHR = 1.81, 95% CI: 1.19–2.71, *p* = 0.005), TNM stage III (SHR = 3.40, 95% CI: 1.90–6.08, *p* < 0.001), poor response to neoadjuvant therapy (SHR = 2.91, 95% CI: 1.99–4.26, *p* < 0.001) and elevated preoperative CA19-9 level (SHR = 1.84, 95% CI: 1.29–2.62, *p* < 0.001) were independent predictors of CSS in elderly RC patients.

**Table 4:** Univariate and multivariate competing risk analyses of predictive factors associated with cancer specific survival in elderly rectal cancer patients received surgical treatment after neoadjuvant chemotherapy.

Variables	Univariate analysis		Multivariate analysis	
	SHR (95% CI)	*p*-value	SHR (95% CI)	*p*-value
Age		< 0.001		0.012
<80	Reference		Reference	
≥80	2.17 (1.44–3.28)		1.76 (1.14–2.74)	
Sex		0.453		
Male	Reference			
Female	0.87 (0.60–1.25)			
GNRI		< 0.001		< 0.001
≥94.6	Reference		Reference	
<94.6	5.57 (3.62–8.57)		3.90 (2.46–6.19)	
ASA score		0.335		
I or II	Reference			
III or IV	1.20 (0.83–1.72)			
Tumor size, mm		0.413		
<50	Reference			
≥50	1.16 (0.82–1.65)			
Distance to the anal verge, cm				
11–15	Reference		Reference	
6–10	0.99 (0.60–1.62)	0.960	0.93 (0.54–1.61)	0.793
0–5	1.63 (1.06–2.50)	0.026	1.29 (0.82–2.06)	0.274
Differentiation grade		< 0.001		0.005
Well or moderate	Reference		Reference	
Poor or worse	3.91 (2.71–5.65)		1.81 (1.19–2.71)	
Histology		0.526		
Adenocarcinoma	Reference			
Mucinous adenocarcinoma or signet-ring cell	1.12 (0.79–1.59)			
Surgical approach		0.601		
Laparoscopic surgery	Reference			
Robotic surgery	1.10 (0.77–1.57)			
yp T stage		0.020		0.435
T1-2	Reference		Reference	
T3-4	1.81 (1.13–2.99)		0.72 (0.32–1.62)	
yp N stage		< 0.001		0.350
N0	Reference		Reference	
N1-2	2.31 (1.63–3.28)		0.75 (0.41–1.38)	
yp TNM stage				
I	Reference		Reference	
II	1.43 (0.75–2.72)	0.280	1.45 (0.78–2.68)	0.240
III	3.35 (1.82–6.15)	< 0.001	3.40 (1.90–6.08)	< 0.001
Neoadjuvant treatment		0.413		
RT	Reference			
CRT	1.16 (0.82–1.66)			
TRG		< 0.001		< 0.001
Good response	Reference		Reference	
Poor response	4.36 (3.03–6.28)		2.91 (1.99–4.26)	
Adjuvant chemotherapy		< 0.001		0.056
Yes	Reference		Reference	
No	2.59 (1.66–4.05)		1.62 (0.99–2.65)	
CEA		< 0.001		0.470
Normal	Reference		Reference	
High	1.75 (1.23–2.50)		1.16 (0.78–1.71)	
CA19-9		< 0.001		< 0.001
Normal	Reference		Reference	
High	1.61 (1.13–2.31)		1.84 (1.29–2.62)	

SHR, subdistribution hazard ration; GNRI, geriatric nutritional risk index; ASA, American society of Aneshesiologists; CRT, chemoradiotherapy; RT, radiotherapy; TRG, tumour regression grade.

### 3.7. Development of cancer-specific mortality prediction models

**Figure 4:**
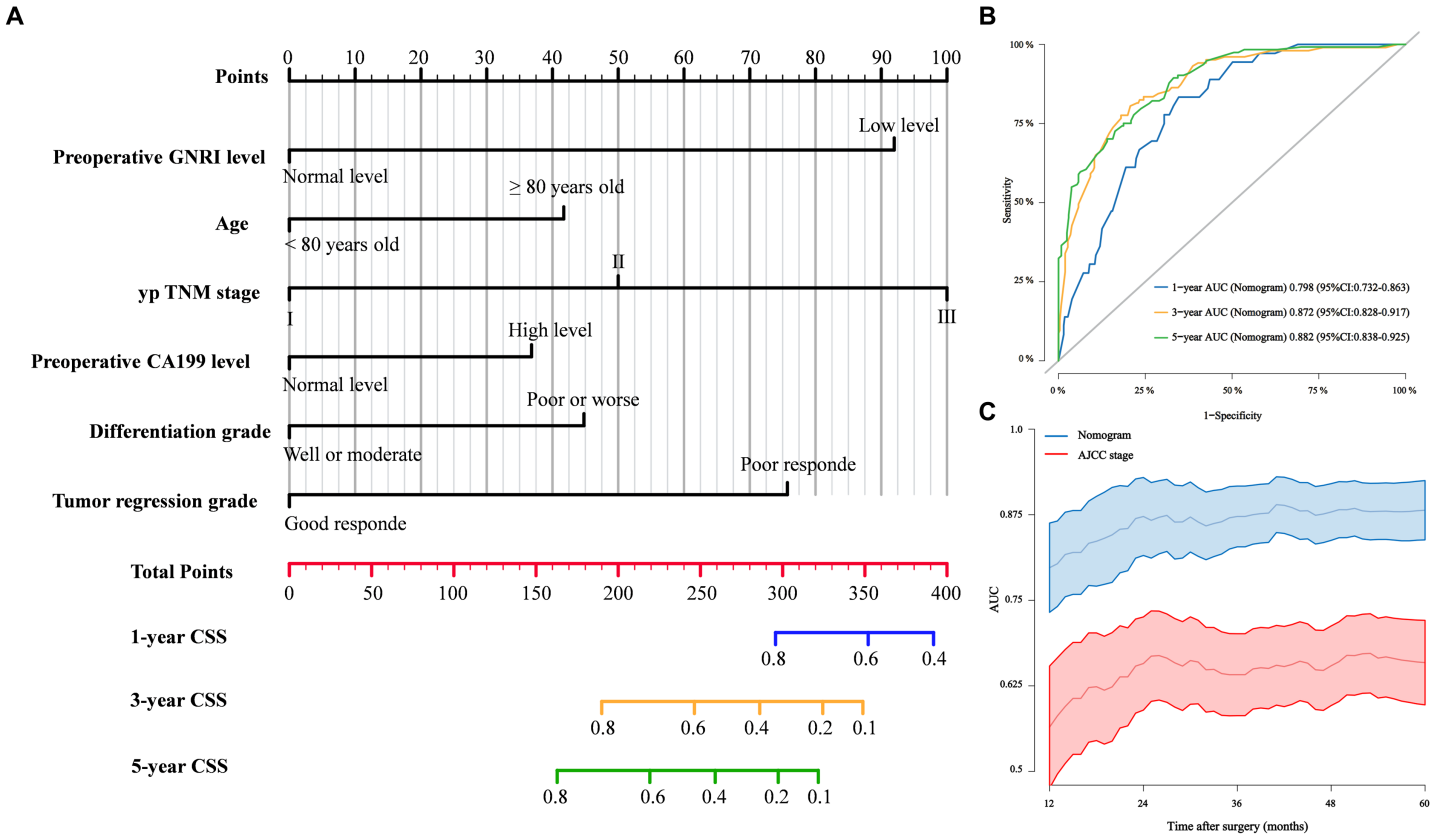
**(A)** Competing risk Model 1 of cancer specific mortality for elderly rectal cancer patients treated with surgery after neoadjuvant therapy; **(B)** 1-, 3-, and 5-year ROC curves comparison of competing risk Model 1; **(C)** Time-dependent ROC curves of competing risk Model 1 and AJCC stage.

Based on the findings of the multivariate competing risk analysis, we initially developed Model 1 to predict the cumulative probabilities of 1-, 3-, and 5-year CSS (Figure 4A). The AUCs of 1-, 3-, and 5-year CSS predicted by Model 1 were 0.798, 0.872 and 0.882, respectively (Figure 4B). Time-dependent ROC curves revealing the discriminatory power of Model 1 and AJCC stage are shown in Figure 4C. Additional models were also developed to compare and validate the predictive value of the preoperative GNRI level. Model 2 contained parameters that were significant in the multivariate analysis except for preoperative GNRI level (Supplementary Figure S6A). Model 3 incorporated the preoperative GNRI level and yp TNM stage (Supplementary Figure S6B). Model 4 contained only the TNM staging system.

### 3.8. Comparison and validation of cancer-specific mortality prediction models

In terms of time-dependent ROC curve analysis, Model 1 exhibited the highest AUC in predicting CSS at various time points postsurgery (Supplementary Figure S7). The 1-, 3-, and 5-year ROC curves for the four models are shown in Supplementary Figure S8. Model 1 accurately predicted patient 1-, 3-, and 5-year CSS with C-indices of 78.1, 81.8, and 80.9%, respectively, which were significantly higher than those of the other models (Supplementary Figure S9). In the DCA plots, Model 1 attained the highest net benefit rate for 1-, 3-, and 5-year CSS prediction (Supplementary Figure S10). ROC curves, calibration plots, and DCA plots were also applied to the validation cohort to predict 1-year CSS, and similar results were obtained (Supplementary Figure S11).

## 4. Discussion

An increasing number of elderly cancer patients require complex and multifaceted care to achieve optimal clinical outcomes from cancer treatment. Assessment of nutritional status is an essential part of the comprehensive management of elderly cancer patients. In the current study, we demonstrated that a low GNRI level was associated with increased postoperative major complications and cancer-specific mortality in RC patients aged ≥70 years who underwent surgical treatment after neoadjuvant therapy. Moreover, based on the analysis results, we constructed a postoperative major complication nomogram and four CSS prediction models for identifying high-risk groups to guide clinical decision-making. These findings support the importance of preoperative nutritional assessment and timely intervention in elderly patients with RC to improve clinical outcomes. As far as we know, the sample size of this study is the largest among other studies assessing nutritional status in elderly RC patients who underwent surgical treatment after neoadjuvant therapy to date.

Recently, several nutritional screening methods, including the Nutritional Literacy Scale, Global Indicator of Malnutrition, and many other serum markers, have been reported for the assessment of nutritional status in elderly cancer patients, but there is not yet a unified standard (18–22). Moreover, most nutritional assessment tools are inconvenient for clinical use and require patient cooperation, which are not appropriate in busy clinical settings. Thus, a simple and effective assessment of a patient’s nutritional status before initiating therapy is essential to identify high-risk patients. The parameters for calculating the GNRI are readily available and can reflect the nutritional status of an organism. In addition, the current weight/ideal weight was set to 1 regardless of how much the patient’s weight exceeded the ideal weight. It is well known that in a narrow pelvis, obese patients can limit the exposure of the surgical field and increase the difficulty of the operation (23). Therefore, overweight patients with RC, who have a special risk of poor prognosis, would not be misclassified as well-conditioned patients. This calculation method is especially suitable for nutritional assessment of RC patients.

In our study, the low-level GNRI group was associated with increased postoperative major complications (OR = 1.903, 95% CI: 1.120–3.233, *p* = 0.017). Our results are consistent with previous research reporting that a low-level GNRI was correlated with increased morbidity in patients with gastric cancer (12) and hepatocellular carcinoma (24). Our results also indicated that the rate of anastomotic leakage was significantly higher in the low-level GNRI patients (7.3% versus 2.6%, *p* = 0.034). This may be because elderly cancer patients with malnutrition often show severe protein-amino acid metabolism disorders and negative nitrogen balance, which lead to reduced healing ability of anastomotic tissues and increase the risk of anastomotic leakage (25). Additionally, hypoalbuminaemia is associated with tissue oedema and impaired collagen synthesis, which can lead to local dyskinesia and increased anastomotic tissue pressure. Therefore, early detection of malnutrition in patients and timely correction can help reduce the incidence of anastomotic leakage. Based on the multivariate analysis results, we further constructed a prediction nomogram quantifying the risk of postoperative major complications in elderly patients with RC who underwent surgical treatment after neoadjuvant therapy. Moreover, the nomogram showed good predictive efficiency in both internal and external verification. This nomogram is expected to facilitate preoperative counselling and be used to develop individualized perioperative strategies to reduce the risk of postoperative complications.

In general, univariate and multivariate Cox regression models are commonly used in survival analysis, with outcomes grouped into death and censored categories. However, in time-to-event analysis, if the patient dies from other causes, the competing events would impede the occurrence of events of interest. In such circumstances, the use of traditional survival analyses would bias the calculation of the cumulative incidence of interest event (26). This phenomenon was most easily seen in elderly patients. Older adults may die from other causes, such as cardiovascular disease, brain stroke, respiratory failure, and accidents, which may preclude the occurrence of cancer-related deaths. Cumulative correlation functions can handle survival data that have multiple outcome events with competing relationships, which could estimate the probability of concurrence of the interest event more accurately (27). Therefore, competition risk analysis was used in prognostic analysis in this study.

In our research, multivariate competing risk analysis revealed that the GNRI could be an indicator of CSS in elderly RC patients (GNRI<94.6, SHR = 3.90, 95% CI: 2.46–6.19, *p* < 0.001). Grinstead et al. found that GNRI <98 was an independent prognostic factor of advanced-stage pancreatic cancer (28). An et al. also showed that compared with patients with GNRI ≥92, patients with GNRI <92 with non-small cell lung cancer had significantly worse CSS (29). Bouillanne et al. first proposed the use of 82, 92 and 98 as the cutoff values of the GNRI to distinguish the nutritional status of elderly medical patients (9). Those cutoff values have been followed in most subsequent studies. However, in this study, X-Tile software was used to determine the best cutoff point for the GNRI. This is primarily because the Bouillanne et al. study focused on elderly medical patients rather than elderly cancer patients (9). Accordingly, potential heterogeneity may exist in the research population. Next, the nutritional baseline of the population may be different in different geographies, and the serum albumin level may differ. Therefore, our cutoff value may be more suitable for distinguishing the nutritional status of elderly patients with RC. Although the cutoff value used in our study varied slightly, the findings consistently support a correlation between preoperative nutritional status and long-term prognosis.

Our results found that preoperative GNRI levels were significantly associated with CSS in RC patients, and the main reasons may be as follows. First, albumin can impact hepatocyte catabolism through the action of proinflammatory cytokines, which are crucial in regulating tumour cell proliferation, apoptosis and invasion (30). A study by Doleman et al. showed that being underweight may increase the risk of disease progression and mortality in RC patients, which may be associated with side effects of cancer cachexia (31). Thus, the GNRI could be employed to predict tumour-related death. To further clarify the predictive value of the GNRI for patient prognosis, four CSS prediction models were constructed separately in our study. Model 1 incorporating the GNRI and other prognostic risk factors provided the best predictive ability. Compared with Model 4, which only included the AJCC staging system, Model 3 significantly improved the prediction performance, although only the GNRI was added. Model 2 included five common independent prognostic risk factors for RC patients, but the discriminative ability was not significantly different from that of Model 3. Moreover, similar results were also obtained in the validation cohort. Thus, assessment of nutritional status has important predictive value for the prognosis of elderly RC patients who undergo surgical treatment after neoadjuvant therapy.

In our study, we found that elderly RC patients with preoperative low-level GNRI accounted for 33.1% (279/842). However, nutritional care is frequently overlooked and undervalued by clinicians despite its importance as a crucial component of the comprehensive assessment of geriatric cancer patients. The nomograms constructed in this study may contribute to identifying high-risk patients with specific clinical outcomes. Based on quantified specific risks, appropriate management of nutritional status and individualized therapies and clinical decision-making should be addressed in this group to improve surgical risk and prognosis.

Our study has several limitations. First, it is a retrospective, nonrandomized, observational study and thus may present some potential selection bias. Second, we did not further evaluate the impact of GNRI before and after neoadjuvant therapy, as well as the dynamic changes in GNRI on clinical outcomes, which may provide more valuable information for clinical practice. Third, due to the short follow-up time of the external cohort, the performance of the competing risk model in predicting 3-year and 5-year CSS was not further validated. Thus, to provide more convincing results, our results still need to be further validated in a multicentre, large sample cohort.

## 5. Conclusion

Our study showed that a preoperative GNRI level < 94.6 was associated with an increased risk of postoperative major complications and cancer-specific mortality in elderly RC patients who underwent surgical treatment after neoadjuvant therapy. The prediction nomograms constructed in this study showed good prediction efficiency in both internal and external verification and could provide effective guidance for patient management.

## Data availability statement

The raw data supporting the conclusions of this article will be made available by the authors, without undue reservation.

## Ethics statement

The Institutional Review Board and Ethical Committee of the First Affiliated Hospital of Xi’an Jiaotong University approved this study (XJTU1AF2020LSL-004). The studies were conducted in accordance with the local legislation and institutional requirements. The participants provided their written informed consent to participate in this study.

## Author contributions

LZ and CH: data collection, formal analysis, investigation, methodology, project administration, software, validation, and writing original. RZL, ZZ, YW, JZ, RHL, and ZL: data curation, formal analysis, investigation, methodology, review, and editing. JS and FS: project administration, validation, review, editing, and supervision. All authors contributed to the article and approved the submitted version.

## Funding

This project was supported by National Natural Science Foundation of China (No. 81870380) and Shaanxi Province Science Foundation (2023-GHYB-13).

## Conflict of interest

The authors declare that the research was conducted in the absence of any commercial or financial relationships that could be construed as a potential conflict of interest.

## Publisher’s note

All claims expressed in this article are solely those of the authors and do not necessarily represent those of their affiliated organizations, or those of the publisher, the editors and the reviewers. Any product that may be evaluated in this article, or claim that may be made by its manufacturer, is not guaranteed or endorsed by the publisher.
