# Supplementary material for: The clinical predictive value of geriatric nutritional risk index in elderly rectal cancer patients received surgical treatment after neoadjuvant therapy
Source: Front Nutr. 2023 Aug 21;10:1237047. doi: 10.3389/fnut.2023.1237047 (PMC10475528; doi:10.3389/fnut.2023.1237047)
Supplement: Supplementary file 1 [file Data_Sheet_1.docx]

**The clinical predictive value of****geriatric nutritional risk index in elderly rectal cancer patients received** **surgical treatment after** **neoadjuvant therapy**

Zhang et al.

(Supplementary Methods)

**The methodologyof propensity score matching**

Each patient was propensity scored in a logistic regression model centered on 11 covariates, including age, sex, ASA score, tumor size, tumor location, differentiation grade, histological type, surgical approach, yp TNM stage, neoadjuvant treatment, and TRG. The matching on the propensity score (1:1) was performed using a nearest neighbour-matching algorithm, with a maximum calliper distance of 0.1 of the standard deviation of the logit of the propensity score.

**The methodologyof competing risk analysis**

Among survival outcomes, rectal cancer-specific mortality was regard as the interest event, while other causes-specific mortality such as cardiovascular cause, respiratory cause, and other reason except that oncological causes was regard as the competing event. The cumulative incidence of events was estimated by the cumulative incidence. Competing risk model was performed in the univariate and multivariate competing risk analysis to find the associations between factors and rectal cancer-specific mortality. And subdistribution hazard ration was estimated to explore the relationships between each variable and interest event. The eventually selected factors were used to build the competing risk nomogram.
