# Supplementary material for: The clinical predictive value of geriatric nutritional risk index in elderly rectal cancer patients received surgical treatment after neoadjuvant therapy
Source: Front Nutr. 2023 Aug 21;10:1237047. doi: 10.3389/fnut.2023.1237047 (PMC10475528; doi:10.3389/fnut.2023.1237047)
Supplement: Supplementary file 2 [file Data_Sheet_2.docx]

**The clinical predictive value of****geriatric nutritional risk index in elderly rectal cancer patients received** **surgical treatment after** **neoadjuvant therapy**

Zhang et al.

(Supplementary Table)

**Supplementary table 1**. Demographic and perioperative characteristics of external validation cohort (n=203)

| Parameters | | | | Results |
| --- | --- | --- | --- | --- |
| Age, yr | | | | 76.5±4.7 |
| Sex | | | |  |
|  | | | Male | 111 (54.7) |
|  | | | Female | 94 (45.3) |
| ASA score | | | |  |
|  | | | I or II | 119 (58.6) |
|  | | | III or IV | 84 (41.4) |
| Tumor size, mm | | | |  |
|  | | | ＜50 | 123 (60.6) |
|  | | | ＞50 | 80 (39.4) |
| Distance to the anal verge, cm | | | |  |
|  | | | 11-15 | 71 (35.0) |
|  | | | 6-10 | 75 (36.9) |
|  | | | 0-5 | 57 (29.1) |
| Differentiation grade | | | |  |
|  | | | Well or moderate | 170 (83.7) |
|  | | | Poor or worse | 33 (16.3) |
| Histology | | | |  |
|  | | | Adenocarcinoma | 156 (76.9) |
|  | | | Mucinous adenocarcinoma or   signet-ring cell | 47 (23.1) |
| Surgical approach | | | |  |
|  | | | Laparoscopic surgery | 141 (69.5) |
|  | | | Robotic surgery | 62 (30.5) |
| yp T stage | | | |  |
|  | | T1-2 | | 42 (20.7) |
|  | | T3-4 | | 161 (79.3) |
| yp N stage | | | |  |
|  | | N0 | | 84 (41.4) |
|  | | N1-2 | | 119 (58.6) |
| yp TNM stage | | | |  |
|  | | | I | 17 (8.4) |
|  | | | II | 67 (43.0) |
|  | | | III | 119 (58.6) |
| Neoadjuvant treatment | | | |  |
|  | | | RT | 74 (36.5) |
|  | | | CRT | 129 (63.5) |
| TRG | | | |  |
|  | | | Good response | 84 (41.4) |
|  | | | Poor response | 119 (58.6) |
| CEA | | | |  |
|  | High | | | 67 (33.0) |
|  | Normal | | | 136 (77.0) |
| CA19-9 | | | |  |
|  | High | | | 37 (18.2) |
|  | Normal | | | 166 (81.8) |
| GNRI | | | |  |
|  | ≥94.6 | | | 131 (64.5) |
|  | <94.6 | | | 72 (35.5) |
| All complications | | | | 53 (26.1) |
| Clavien-Dindo grade ≥ II | | | | 41 (20.2) |
| GNRI:geriatric nutritional risk index; ASA: American society of Aneshesiologists; CRT: chemoradiotherapy; RT: radiotherapy; TGR: tumour regression grade. | | | | |
