# Supplementary material for: The clinical predictive value of geriatric nutritional risk index in elderly rectal cancer patients received surgical treatment after neoadjuvant therapy
Source: Front Nutr. 2023 Aug 21;10:1237047. doi: 10.3389/fnut.2023.1237047 (PMC10475528; doi:10.3389/fnut.2023.1237047)
Supplement: Supplementary file 3 [file Data_Sheet_3.docx]

**The clinical predictive value of****geriatric nutritional risk index in elderly rectal cancer patients received** **surgical treatment after** **neoadjuvant therapy**

Zhang et al.

(Supplementary Figures)


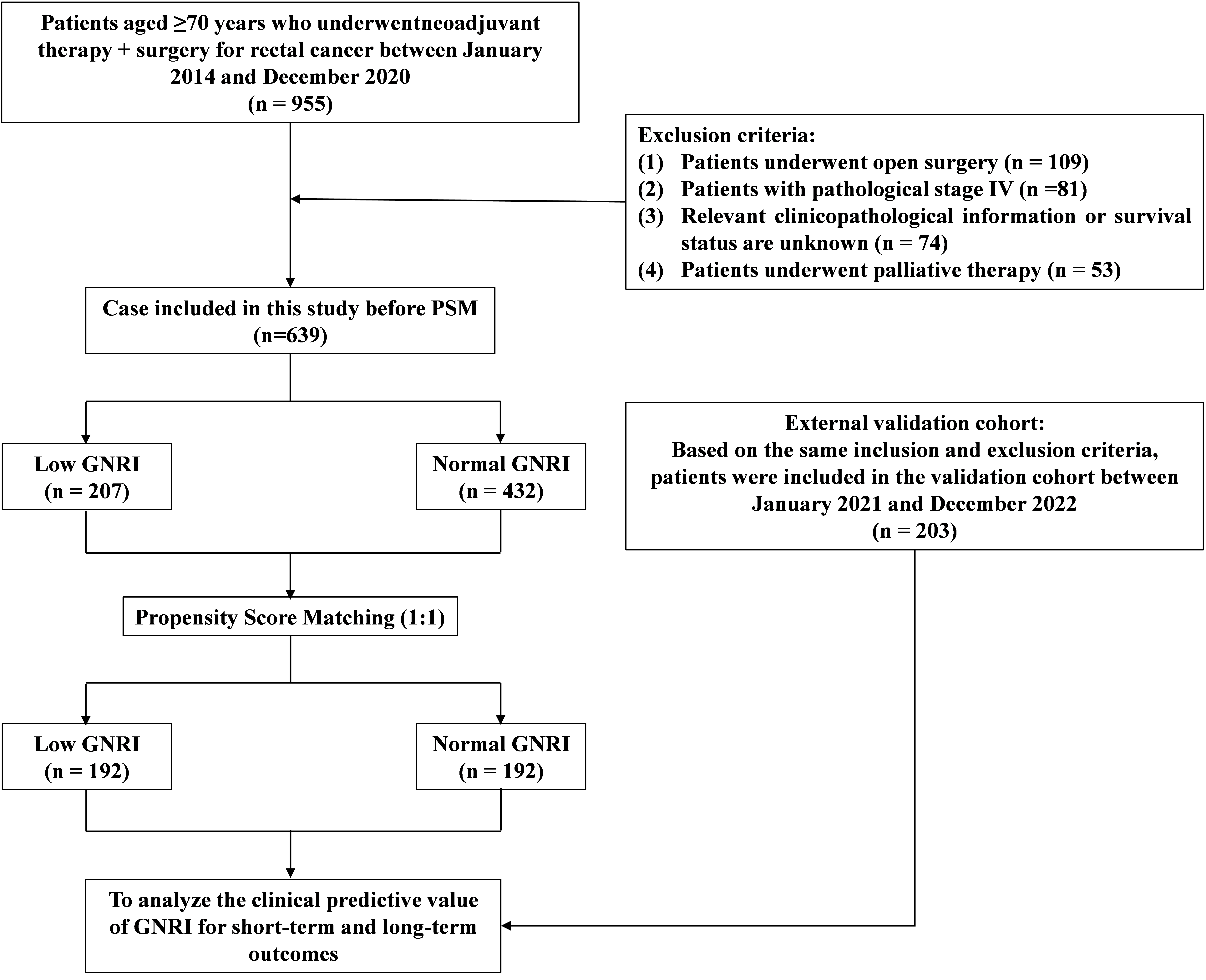


**Supplementary Figure. 1** The inclusion criteria flowchart of study patients. GNRI:geriatric nutritional risk index; PSM:propensity score matching.


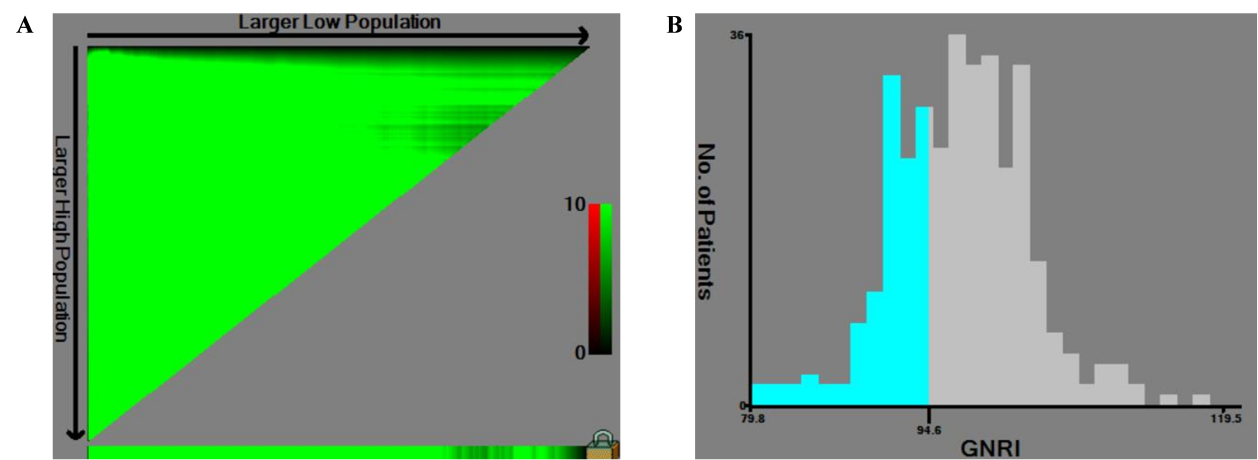


**Supplementary Figure. 2** Analysis of the best-cutoff point of preoperative GNRI level using X-tile program. A X-tile plot of GNRI; B the cutoff point was highlighted using a histogram of the entire patients.


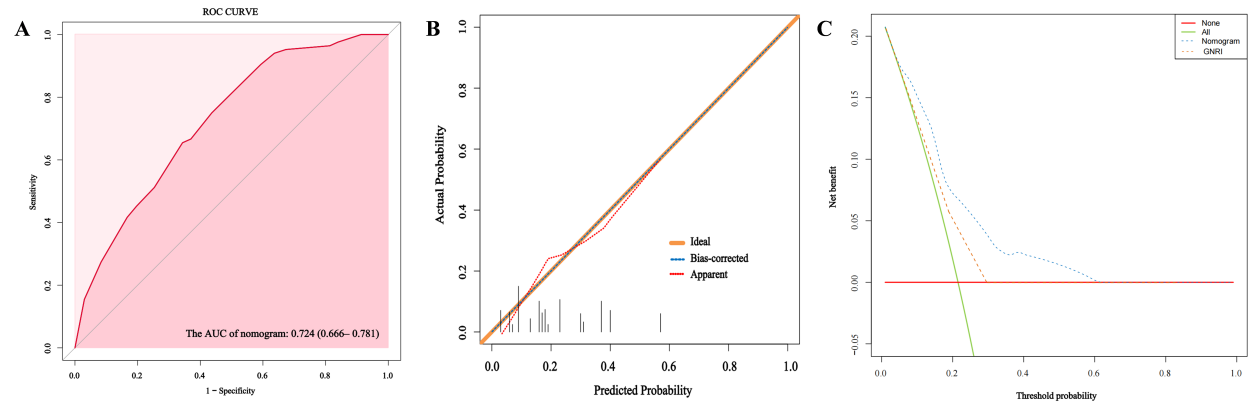


**Supplementary Figure. 3** The ROC curve (A), calibration curves (B) and DCA curves (C) for the postoperative complication nomogram in external validation cohort.

of the nomogram.


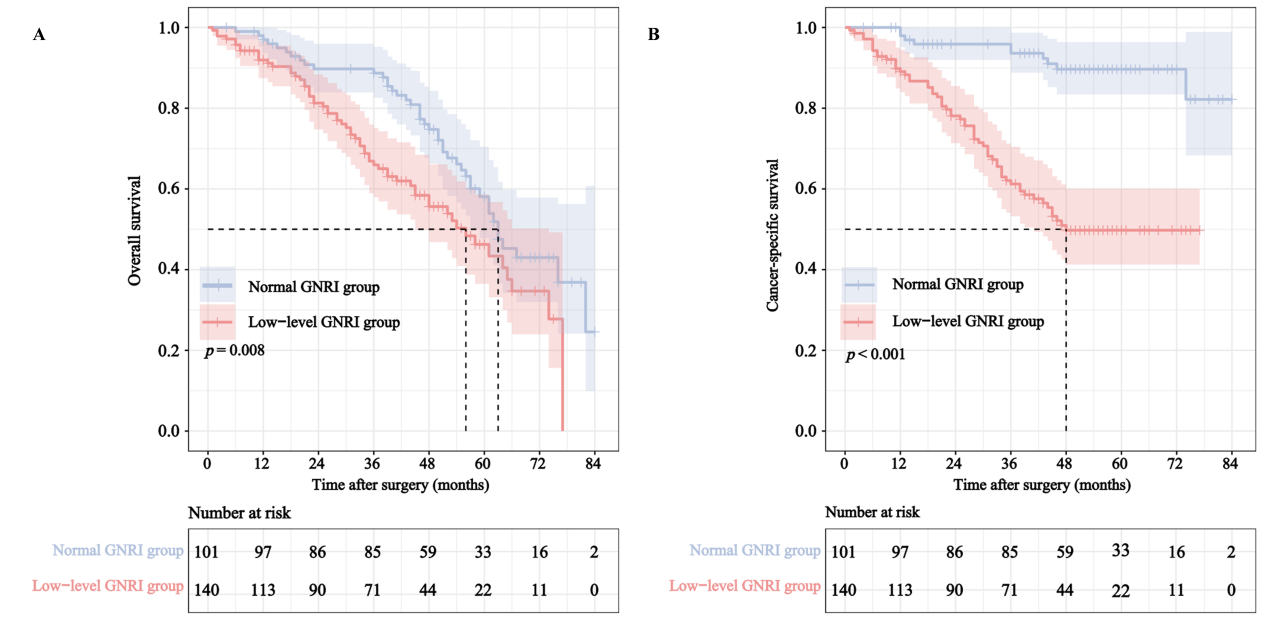


**Supplementary Figure. 4** Overall survival curves (A) and cancer-specific curves (B) of rectal cancer patients aged 70 to 80 years with different GNRI levels.


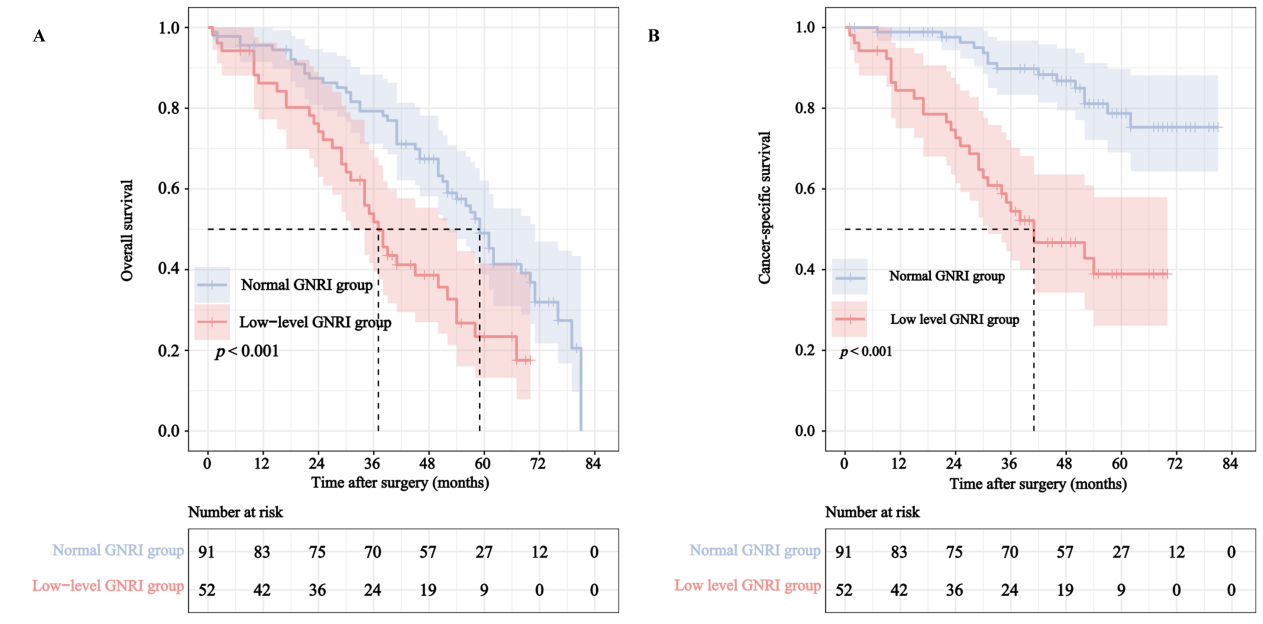


**Supplementary Figure. 5** Overall survival curves (A) and cancer-specific curves (B) of rectal cancer patients over 80 years of age with different GNRI levels.


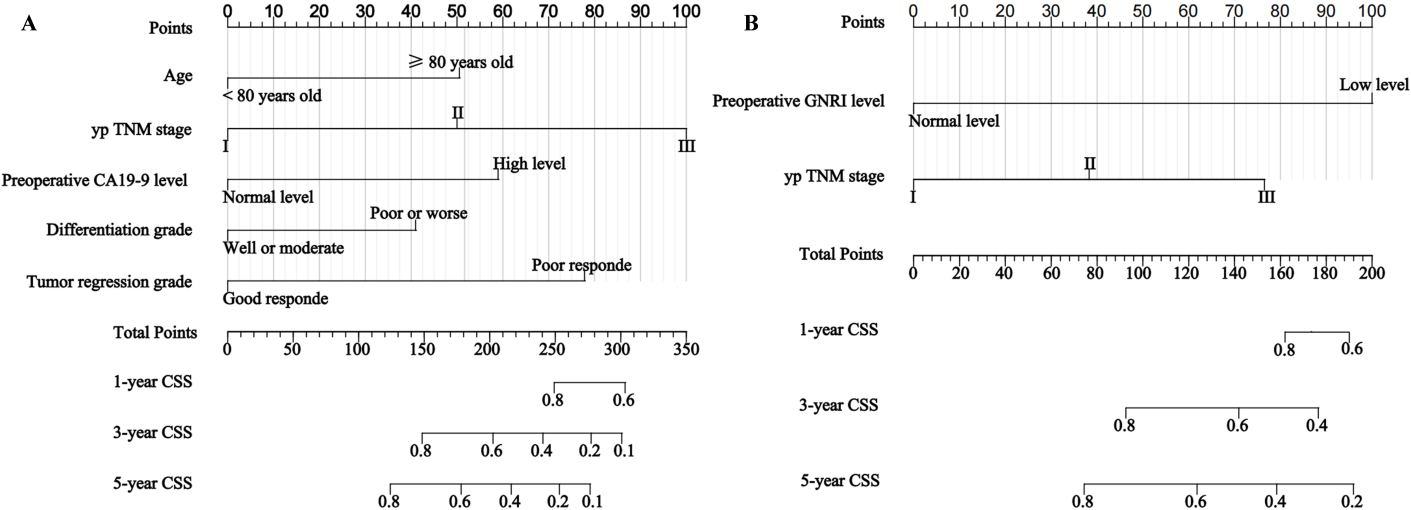


**Supplementary Figure. 6** (A) Nomogram model 2 and (B) Nomogram model 3.


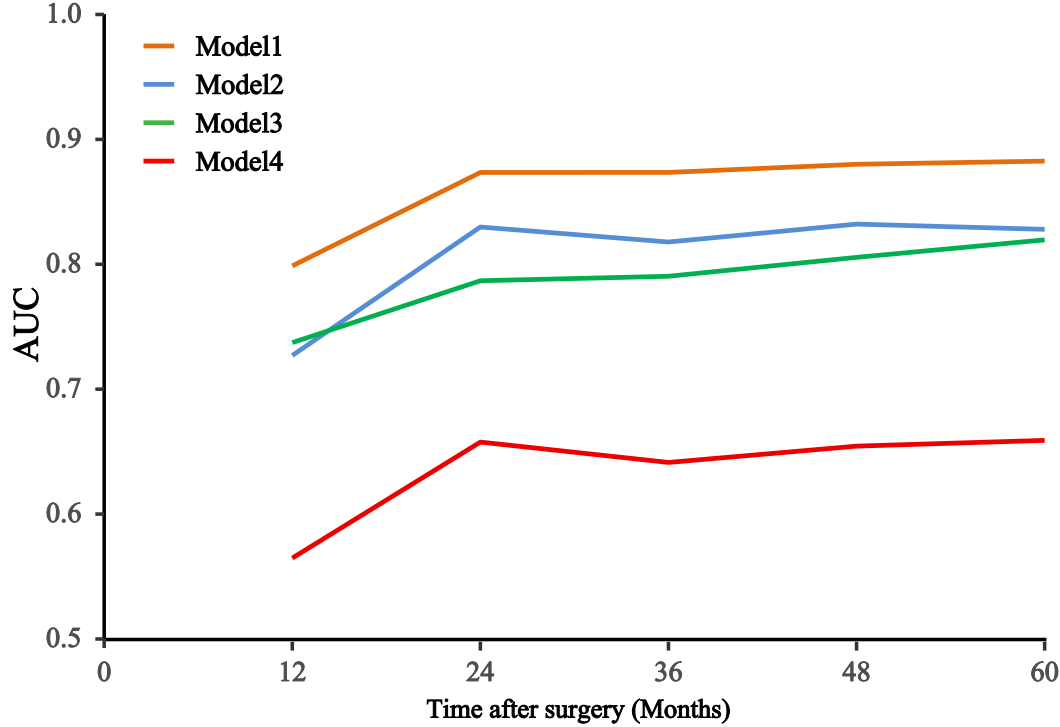


**Supplementary Figure. 7** Time-dependent ROC curves of different cancer-specific death prediction models.


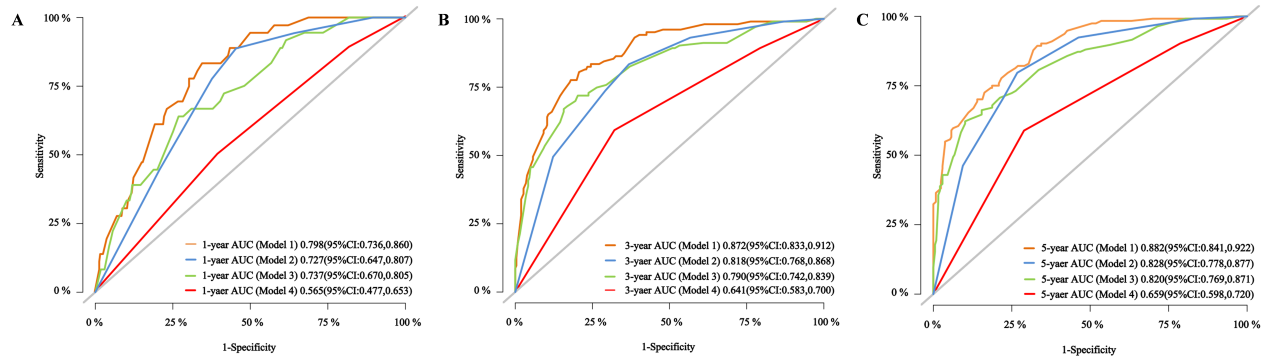


**Supplementary Figure. 8** ROC curves for different models in the prediction of the cancer-specific death of patients at 1- (A), 3- (B) and 5-year (C) point.


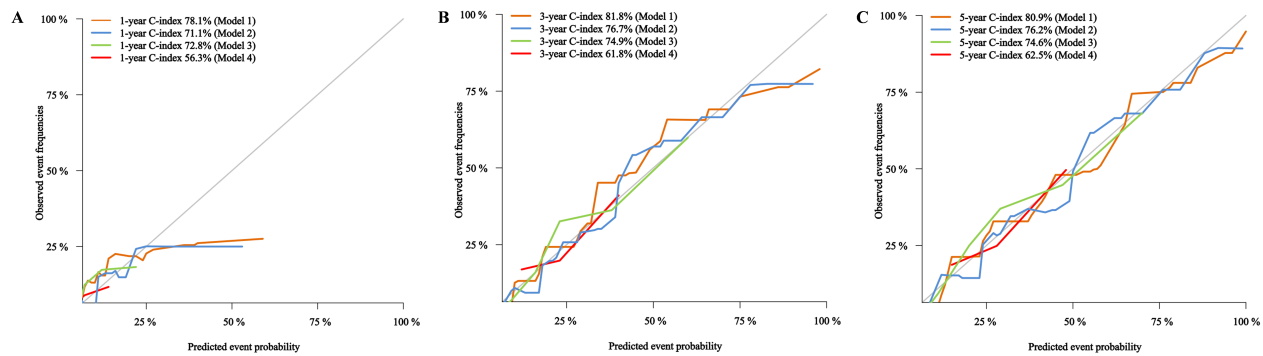


**Supplementary Figure. 9** Calibration curves for different models in the prediction of the cancer-specific death of patients at 1- (A), 3- (B) and 5-year (C) point.


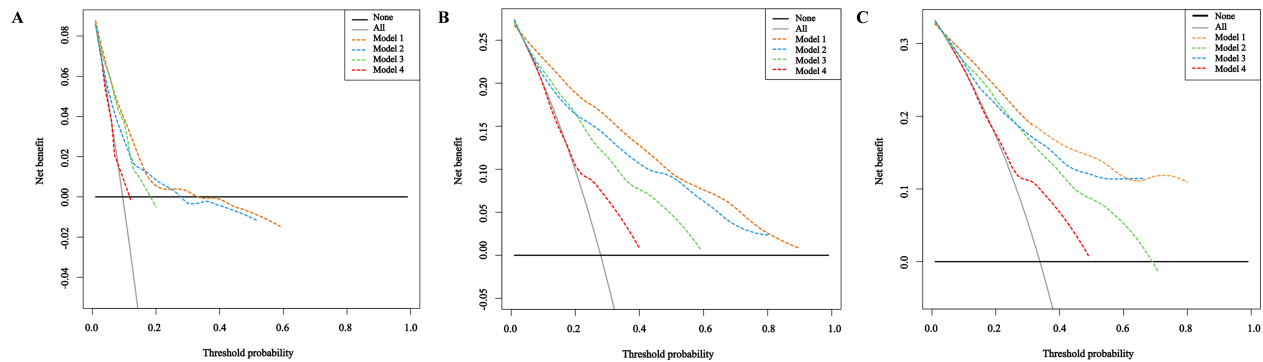


**Supplementary Figure. 10** Decision curve analysis for different models in the prediction of the cancer-specific death of patients 1- (A), 3- (B) and 5-year (C) point.


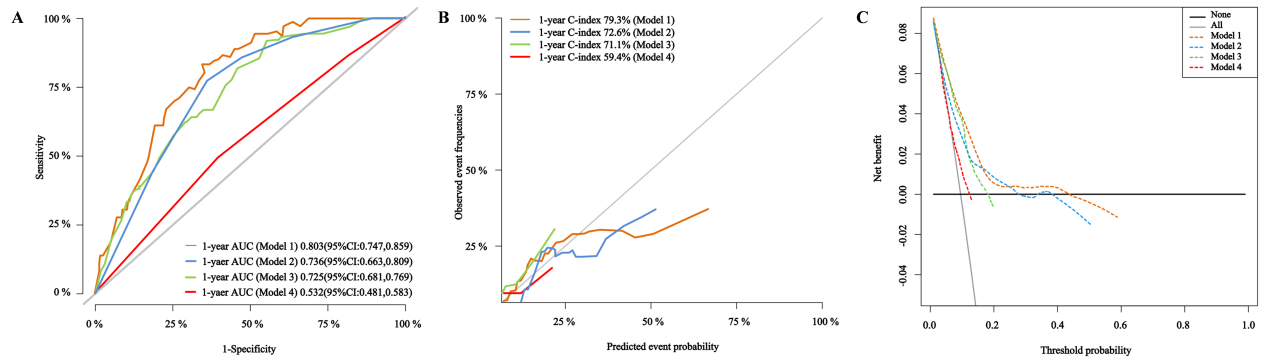


**Supplementary Figure. 11** The 1-year ROC curves (A), calibration curves (B) and DCA curves (C) for different models in external validation cohort.
